# Editorial: Neurotoxins in Alzheimer's disease and other dementias

**DOI:** 10.3389/fnagi.2024.1363466

**Published:** 2024-01-11

**Authors:** Alexandre Henriques, Philippe Léon Louis Poindron, Binosha Fernando, Kevin N. Hascup

**Affiliations:** ^1^Neuro-Sys, Gardanne, France; ^2^Centre of Excellence for Alzheimer's Disease Research and Care School of Medical and Health Sciences, Edith Cowan University, SNRI, Nedlands, WA, Australia; ^3^Department of Neurology, Pharmacology, and Medical Microbiology, Immunology and Cell Biology, Center for Alzheimer's Research and Treatment, Southern Illinois University School of Medicine, Springfield, IL, United States

**Keywords:** Alzheimer's disease, dementia, neurotoxicants, N-nitrosodimethylamine (NDMA), copper, apolipoprotein E4, Heat Shock Protein 90 (Hsp90), metabolomics

Alzheimer's disease (AD) and other dementias are neurodegenerative disorders characterized by a progressive decline in cognition and independence from activities of daily living. Dementia is multifactorial with numerous risk factors including age, genes, molecules, lifestyle, and environmental contributions to disease onset and progression. In recent years, an emerging focus on neurotoxins has added a new layer of complexity to our understanding of dementia. This editorial aims to discuss recent updates regarding the role of neurotoxins in the pathogenesis of dementia.

## Copper's double-edged sword

Copper, a trace element crucial for various physiological processes, can accumulate in the brain over time. The article “Copper and cuproptosis: New therapeutic approaches for Alzheimer's disease” reviews compelling evidence linking excessive free copper deposition to cognitive decline in individuals with AD (Li et al.). Elevated free copper levels in the serum and brain of AD patients lead to reduced antioxidant defenses and mitochondrial dysfunction, setting the stage for neurodegeneration. Moreover, the accumulation triggers a specific form of cell death, known as copper-dependent cell death (cuproptosis). This revelation prompts a reevaluation of the intricate relationship between copper dysregulation and the progression of AD. The efficacy and safety of interventions, such as copper chelators, lipid peroxidation inhibitors, and antioxidants, are also explored. These treatments aim to restore copper equilibrium and prevent copper-induced cell death in AD cases, offering a glimmer of hope in the pursuit of pharmaceutical interventions to address copper dysregulation.

## NDMA's stealthy entry into the Alzheimer's puzzle

N-nitrosodimethylamine (NDMA), an environmental and food contaminant, emerges as an unsuspected player in the neurotoxin repertoire considering it is a widely recognized carcinogen. The study “Association of N-nitrosodimethylamine exposure with cognitive impairment based on the clues of mice and humans” investigates the concentration of NDMA in foods and its effects on both murine and human cognition (Liu et al.). The findings suggest an association between NDMA and cognitive decline, providing a new perspective on the environmental factors that may contribute to AD. This study opens avenues for further research into the potential link between environmental carcinogens and neurodegenerative disorders.

## Apolipoprotein E4: unraveling the genetic thread

Apolipoprotein E4 (ApoE4) is the largest genetic risk variant for developing late-onset Alzheimer's disease. The protein plays an essential role in providing cholesterol and other lipids for neuronal utilization. “Neuronal ApoE4 in Alzheimer's disease and potential therapeutic targets” explores the role of ApoE4 in the development and progression of AD (Zhang et al.). This review emphasizes how ApoE4 in neurons induces amyloid beta (Aβ) and tau protein pathologies, leading to neuroinflammation and neuronal damage. Understanding the pathophysiology of neuronal ApoE4 becomes crucial in developing targeted therapeutic strategies to mitigate the risk of AD development.

## Hsp90 modulators: a molecular approach

The presence of misfolded proteins in neurodegenerative diseases brings attention toward the dysregulation of molecular chaperones. “Protection against Aβ-induced neuronal damage by KU-32: PDHKI inhibition as important target” introduces novobiocin, a modulator at the C-terminal ATP-binding site of Heat Shock Protein 90 (Hsp90) (Pal et al.). The study explores the effects of novobiocin analogs, specifically focusing on KU-32, in protecting neurons from Aβ-induced death. This approach not only highlights potential therapeutic avenues but also sheds light on the intricate relationship between protein misfolding and AD pathology.

## Metabolomics: elucidating small molecules impact

Metabolomics provides a powerful tool for comprehensively measuring low-molecular-weight molecules from biological samples. The study “Metabolomic profiling of CSF and blood serum elucidates general and sex-specific patterns for mild cognitive impairment and Alzheimer's disease patients” quantifies metabolites in cerebrospinal fluid and serum, revealing significant alterations associated with dementia (Berezhnoy et al.). Reduced levels of ketone bodies, branched-chain amino acids, and alterations in valine degradation pathways in AD patients highlight the metabolic shifts in the disease. The study underscores the potential of metabolomics in uncovering novel biomarkers and therapeutic targets for AD.

## Aβ_1 − 42_ oligomers: enticing seizure susceptibility

Hyperactive neuronal networks and seizures are present in AD patients and genetic animal models. Although Aβ is an established biomarker for AD, its role in seizure susceptibility remains unclear. “The intracerebral injection of Aβ_1 − 42_ oligomers does not invariably alter seizure susceptibility in mice” examines the effects of oligomeric Aβ_1 − 42_ on seizure susceptibility in NMRI outbred mice (Vande Vyver et al.). While *ex vivo* slice work highlights a biological role for Aβ_1 − 42_ on hippocampal hyperactivity, no effects after intracerebral injection were observed *in vivo*. The *in vivo* outcomes diverge from other research findings, underscoring the significance of conducting comprehensive biophysical characterizations of Aβ_1 − 42_ for meaningful cross study comparisons.

## Conclusion

In conclusion, the neurotoxin landscape in Alzheimer's disease is multifaceted, involving elements of copper dysregulation, environmental contaminants, genetic factors, molecular chaperones, metabolic shifts, and Aβ_1 − 42_ oligomers. This editorial aims to coalesce these diverse areas of research into a comprehensive narrative that contributes to our evolving understanding of AD. As the puzzle pieces come together, the hope is that these insights will pave the way for targeted interventions and therapeutic strategies in the pursuit of optimized precision medicine for those suffering from all forms of dementia.

## Author contributions

AH: Writing – review & editing. PP: Writing – review & editing. BF: Writing – review & editing. KH: Writing – original draft.

